# Pressure pain sensitivity maps of the neck-shoulder and the low back regions in men and women

**DOI:** 10.1186/1471-2474-11-234

**Published:** 2010-10-12

**Authors:** Asbjørn T Binderup, Lars Arendt-Nielsen, Pascal Madeleine

**Affiliations:** 1 Laboratory for Ergonomics and Work-related Disorders, Center for Sensory-Motor Interaction (SMI), Department of Health Science and Technology, Aalborg University, Aalborg, Denmark

## Abstract

**Background:**

Musculoskeletal pain in the low back and neck-shoulder regions is a major problem among the working population all over the world. The prevalence of musculoskeletal pain is found to be higher among women. Women also have lower pressure pain thresholds (PPTs) than men. Pressure pain topography aims at mapping the spatial distribution of PPT within a muscle in an attempt to track changes in mechanical sensitivity. In order to assess gender differences in the pain topography, it is necessary to map the distribution in both healthy men and women. The aim of this study was to assess PPT maps from the cervico-thoracic and lumbar regions in men and women.

**Methods:**

Eleven men and eleven women without any known musculoskeletal disorders participated in the study. PPT was measured twice at 36 points over the trapezius muscle of the dominant arm, at 36 points over the trapezius muscle on the contralateral side and at 12 points over the spine between the left and right trapezius. Further, 11 points were measured over the erector spinae muscle on the left side of the spine between the first and the fifth lumbar vertebrae, 11 on the right side and 5 points on the spine itself. The measurements on each trapezius muscle were divided according to anatomical subdivisions. Three-way and two-way ANOVAs were used to analyse the differences in PPTs with the following factors: gender, locations and sub-divisions (only for cervico-thoracic region).

**Results:**

There were no differences between left and right side in neither the cervico-thoracic nor the lumbar region, but there were (large effect) differences between the subdivisions in the trapezius with the lowest values in the upper part (P < 0.001; partial η^2 ^= 0.19). Women had (small effect) lower PPT in both cervico-thoracic and lumbar regions (P ≤ 0.001; partial η^2 ^= 0.02 for both regions), but gender had no effect on neither location nor subdivisions.

**Conclusions:**

The pain topography was not found to be different between genders in the cervico-thoracic and lumbar regions. This study can be used as basis for further clinical studies on musculoskeletal disorders.

## Background

Chronic musculoskeletal pain causes reduced quality of life with loss of work and social networks [1]. The use of quantitative diagnostic tests for mechanistic evaluation of musculoskeletal pain is important as many mechanisms such as soreness, hyperalgesia and spreading of pain/sensitization are involved [2]. Chronic musculoskeletal pain is most often reported from the low back and shoulder regions [3]. Further, a recent epidemiological study has emphasized a correlation between low back and neck pain in adults [4]. Moreover, the prevalence of musculoskeletal disorders accompanied by pain is higher in women compared with men [5]. Thus, the study of mechanical sensitivity and the spread of pain in the neck-shoulder (cervico-thoracic) and low back (lumbar) regions can help characterizing these pain conditions in men and women.

Pressure pain thresholds (PPTs) are found to be reduced in whiplash [6,7] and work-related musculoskeletal pain [8-10]. In addition PPTs are found to be lower in women as compared with men [6,11-16], and the cause of this difference is not fully understood [14,17] but assumed to include both physiological [18], cultural [19] and psychological factors [20].

Pressure pain algometry is a reliable technique [21] for the assessment of mechanical pain sensitivity of deep somatic structures. However, PPTs are found to vary along a muscle [22,23]. Hence, recordings of PPTs at many locations over a body region can delineate the extent of hyperalgesia [24]. Recently, PPT mapping of the trapezius muscle has underlined spatial pain sensitivity differences among subdivisions of the muscle [24] indicating sensory partitioning [25].

The aims of this study were 1) to assess PPT maps from the left and right trapezius muscles as well as the lumbar region, including the spinal processes from the first to the fifth lumbar vertebra and the extensor spinae muscles on both sides of the spine and 2) to investigate gender differences in PPT maps. We hypothesized lower PPT in women compared with men and symmetry in the topography of the PPT maps among genders.

## Methods

### Subjects

Twenty-two healthy volunteers participated in this study. For the men (N = 11) the average age (± standard deviation) was 23.4 ± 2.5 years, height was 181.2 ± 6.5 cm, body mass was 75.1 ± 8.4 kg and body mass index was 22.9 ± 2.3 kg/m^2^. For the women (N = 11) the average age was 23.9 ± 3.4 years, height was 167.1 ± 8.0 cm, body mass was 62.5 ± 9.7 kg and body mass index was 22.3 ± 2.6 kg/m^2^. One person in each gender group was left handed while the remaining ten were right handed. None of the subjects reported pain or soreness in the neck-shoulder or low back regions prior to entering the study. The study was approved by the local ethics committee (No. N-20070004) and was conducted in accordance with the Declaration of Helsinki. All subjects signed an informed consent upon entering the study.

### Experimental protocol

**Figure 1:**
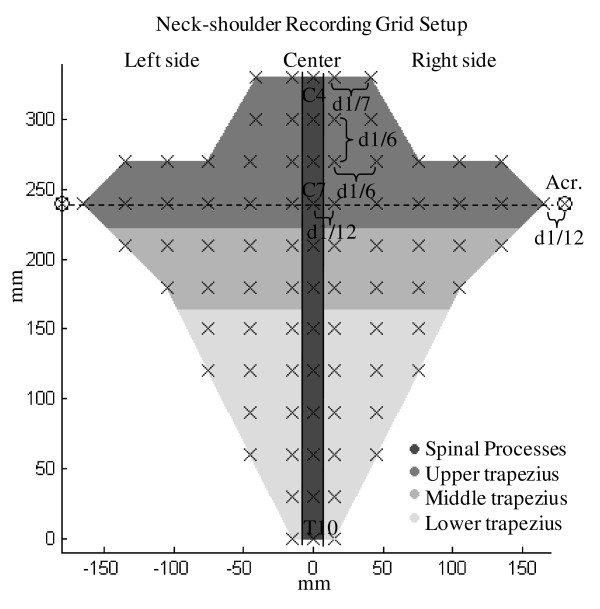
**Schematic representation of the neck-shoulder PPT recording grid (84 points)**. All distances between adjacent points were based on an anthropometric measure (the distance (d1) between the seventh cervical vertebrae (C7) and acromion (Acr.)). The cervico-thoracic region was divided by location (left side, center and right side) and trapezius subdivisions (upper, middle, lower trapezius).

**Figure 2:**
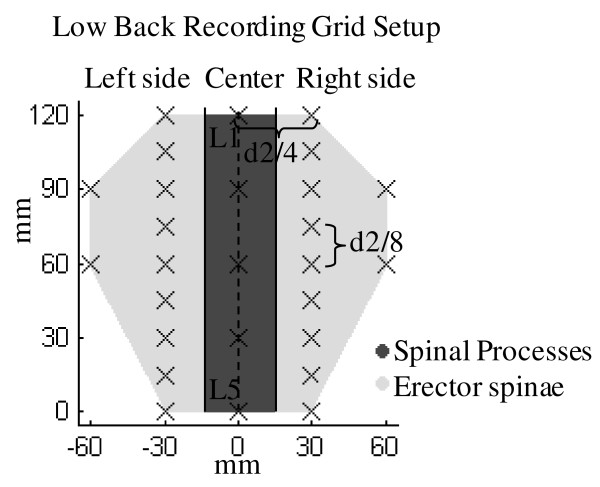
**Schematic representation of the low back PPT recording grid (27 points)**. All distances between adjacent points were based on an anthropometric measure (the distance (d2) between the first (L1) and fifth (L5) lumbar vertebrae). The lumbar region was divided by location (left side, center and right side).

The PPT measurements were performed in one session lasting approximately two hours. For the cervico-thoracic region, PPTs were measured at 36 points over the trapezius muscle of the dominant hand, at 36 points on the contralateral side and at 12 points on the spinal processes from the fourth cervical to the tenth thoracic vertebrae giving a total of 84 measurement points (fig. 1). The distance between two adjacent points was based on the distance between the seventh cervical vertebrae (C7) and the acromion bone of the subject (d1), which on average was found to be 17.9 ± 1.1 cm for men and 14.2 ± 1.3 cm for women. Between most points this distance was one sixth of d1, except for the points in the neck region where, due to muscle size restraints, the horizontal distance was one seventh of d1. Further, all points on the spine were distanced horizontally one twelfth of d1 to adjacent points. For the lumbar region, PPT was measured at 27 points; five points located on the spinal processes between the first (L1) and the fifth (L5) lumbar vertebrae and 22 points located on the erector spinae muscles on each side of the spine (see fig. 2). The distance between adjacent points in this region was based on the distance between the L1 and L5 (d2), which on average was 14.3 ± 2.8 cm for men and 12.5 ± 0.9 cm for women. All horizontal distances were one fourth of d2 and vertical distances were one eighth of d2. A hand-held algometer (Somedic^® ^Algometer type 2, Sweden) with a 1 cm^2 ^wide rubber tip was used for the PPT measurements. The algometer was pressed against the skin in a perpendicular angle at a constant slope of 30 kPa/s. For cervico-thoracic measurements the subjects were sitting, slightly bending forward with their chest in contact with the backrest of the chair. For lumbar measurements the subjects were lying on their stomach on a bed. The subjects were holding a button which they should press to lock the display of the algometer and thus mark when the pain threshold was reached. The subjects were instructed to press the button at the first feeling of change from pressure to pain. Each point was measured twice over two rounds. In each round the measurement order was randomized between different column and row patterns starting either at outer points and going inward or at inner points and going outward. A third PPT measurement was made if the coefficient of variance of the two first measurements at a specific point was above 0.2. The mean value of the two measurements with the lowest variance was used as the PPT value of this point. For points with a coefficient of variance below 0.2 the PPT value was computed as the mean of the first two measurements. One series of measurements would take at least 10 minutes. All PPT measurements were performed by the same examiner. The PPT data were interpolated using inversed distance interpolation by a factor of 2 with Franke and Nielson weightings [26] to display the topographical pain distribution. The map of the cervico-thoracic region was standardized to match the size of a subject with a C7-acromion distance of 18 cm, and the map of the lumbar region was standardized to match the size of a subject with a L1-L5 distance of 12 cm.

### Data analysis

Both absolute PPT values and normalized PPT values were analyzed. For the normalization procedure for each subject we divided all absolute PPT values by the mean PPT value. The measurements were made alternately on the left and right side for subjects with dominant left hand. For the cervico-thoracic region the 36 measurement points were divided according to gender, location (left side, right side and center/spinal processes) and subdivisions, roughly matching the three anatomical subdivisions of the muscle (upper, middle, lower trapezius) along with a fourth part corresponding to the points located on the spinal processes (see fig. 1). A three-way analysis of variance (3-way ANOVA) with factors: gender, location and subdivision was used for the cervico-thoracic region. For the lumbar region the 27 measurements points were only divided according to gender and location (left side, right side and center/spinal processes, see fig. 2). A 2-way ANOVA with factors: gender and location was used for the lumbar region. To evaluate the intra-examiner variability, we computed the correlation (Pearson, two-tailed) between the first and the second round of PPT measurements (according to point location, not measurement order) used to compute the mean PPT value for each subject. For the statistical analysis P < 0.05 was considered significant. In order to report effect size we computed partial η^2 ^(SS_factor_/(SS_factor _+ SS_error_) where SS_factor _is the variation attributable to the factor and SS_error _is the error variation). Post hoc tests were made using Bonferroni adjustment. All results are presented as mean ± standard deviation and ranges.

## Results

### Intra-examiner variability

There was significant correlation for all subjects between the two rounds of PPT measurements for both the cervico-thoracic region (correlation coefficient: 0.82 ± 0.07) and the lumbar region (correlation coefficient: 0.91 ± 0.07).

### Cervico-thoracic region and gender

**Figure 3:**
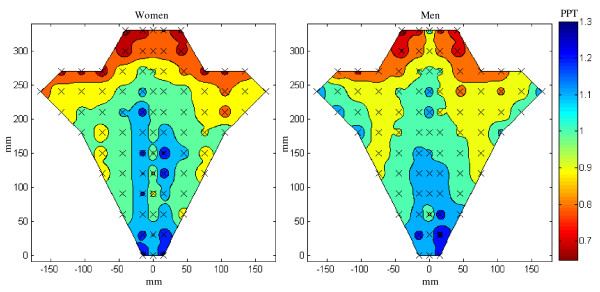
**Normalized pressure pain threshold maps of the neck-shoulder region for women (N = 11) and men (N = 11)**. Note the symmetry along the spine and that the most sensitive part of the muscles is the upper region of the trapezius in both genders.

Men were found to have significantly higher (small effect) PPT than women (357.1 ± 101 vs. 328.9 ± 121.6 kPa; range: 134 - 980 vs. 99 - 920 kPa; P < 0.001, partial η^2 ^= 0.02). The individually normalized PPT increased significantly (large effect) from the upper to lower trapezius (0.88 ± 0.20 vs. 1.11 ± 0.21; range: 0.30 - 2.08 vs. 0.34 - 2.00; P < 0.001, partial η^2 ^= 0.19) independently of the gender or the left/right side (see table 1 and fig. 3).

**Table 1:** Pairwise comparisons of absolute and normalized pressure pain thresholds (PPT) from the cervico-thoracic region.

Subdivision	Absolute PPT (kPa)	Normalized PPT
Upper trapezius	295.2 ± 95.9 *,†,‡ [99 - 981]	0.88 ± 0.20 *,†,‡ [0.30 - 2.08]

Middle trapezius	347.5 ± 103.5 *, • [138 - 745]	1.04 ± 0.18 *, • [0.49-1.70]

Lower trapezius	373.0 ± 121.1 †, • [127 - 920]	1.11 ± 0.21 †, •, Δ [0.34-2.00]

Spinal processes	369.6 ± 116.5 ‡ [156 - 689]	1.04 ± 0.17 ‡,Δ [0.55 - 1.58]

The results of the pairwise comparisons of the PPT values (mean ± SD and [range]). Symbols mark significant difference (P < 0.05) between respective pair of absolute values (*: upper and middle trapezius, †: upper and lower trapezius, ‡: upper trapezius and spinal processes, •: middle and lower trapezius) and normalized values (*: upper and middle trapezius, †: upper and lower trapezius, ‡: upper trapezius and spinal processes, •: middle and lower trapezius, Δ: lower trapezius and spinal processes).

### Lumbar region and gender

**Figure 4:**
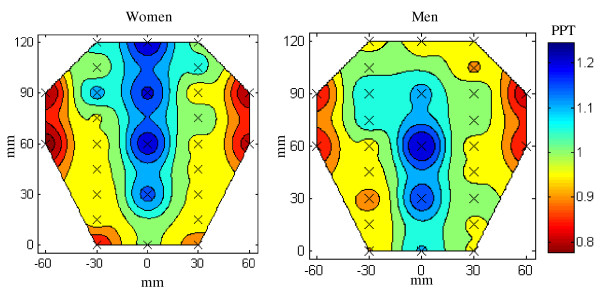
**Normalized pressure pain threshold maps of the low back region for women (N = 11) and men (N = 11)**. Note the lowest sensitivity along the spine. Also note the symmetry along the spine in both genders.

Similar to the cervico-thoracic region, men had significantly higher (small effect) PPT than women (506.1 ± 322.8 vs. 428.2 ± 136.9 kPa; range: 165 - 1926 vs. 213 - 1300 kPa; P = 0.001, partial η^2 ^= 0.02). The normalized PPT values were significantly higher (moderate effect) for the spinal processes than for the left/right side (1.15 ± 0.29 vs. 0.97 ± 0.15; range: 0.70 - 2.08 vs. 0.52 - 1.60; P < 0.001, partial η^2 ^= 0.12) independently of gender (see table 2 and fig. 4).

**Table 2:** Pairwise comparisons of absolute and normalized pressure pain thresholds (PPT) from the lumbar region.

Location	Absolute PPT (kPa)	Normalized PPT
Left side	447.5 ± 229.3 * [167 - 1596]	0.97 ± 0.16 * [0.55 - 1.60]

Spinal processes	536.0 ± 269.5 *,† [165 - 1645]	1.15 ± 0.29 *,† [0.70 - 2.08]

Right side	455.5 ± 258.2 † [181 - 1926]	0.97 ± 0.15 † [0.52 - 1.44]

The results of the pairwise comparisons of the PPT values (mean ± SD and [range]). Symbols mark significant difference (P < 0.05) between respective pair of absolute values (*: left side and spinal processes, †: right side and spinal processes) and normalized values (*: left side and spinal processes, †: right side and spinal processes).

## Discussion

The study revealed heterogeneity in the topography of the pressure pain sensitivity maps in both the cervico-thoracic and the lumbar regions. The pain topography was symmetrical along the spine for both cervico-thoracic and lumbar regions, and there was no difference between left and right side for the cervico-thoracic region while differences were found for the lumbar region. The lower part of the trapezius was the least sensitive to pressure pain while the upper part was the most sensitive. Further, the measurements from the lumbar region showed that musculo-tendinous tissue was more sensitive to pressure than the bones. These proportional topographic differences were observed in both men and women. Furthermore, despite its low population size and small effect size, the present study confirmed that women have lower PPTs than men.

### Gender differences

The present study despite its limitations (see Methodological limitations) showed that women had lower absolute PPT values than men in line with previous studies [6,11-16]. Differences were also found in the lumbar region supporting previous results reported in the erector spinae muscle group [27,28] while no gender differences are reported in the masseter muscle [29] suggesting that gender differences in pressure pain sensitivity may be muscle specific. There is to date no clear understanding of these gender differences [14,17], but the causes are most likely multi-factorial including physiological (e.g. sub-cutaneous fat, muscle size), cultural and psychological components [18-20]. For example, the fact that gender differences are reported to be related to levels of gonadal hormones [30] has been challenged by a recent paper showing no relation between menstruation cycle and pressure pain sensation in the trapezius muscle [31].

The pain sensitivity maps could be influenced by the probe size, but as the receptive fields of the muscle nociceptors in both men and women have been found to be far greater (>3 cm^2^) [32] than the size of the probe used (1 cm^2^), this should not be of major importance. Though, the relative size difference between the muscle and the probe might have an influence as it has been shown that an increase in the probe size will result in a lower PPT when applied to a trapezius muscle due to spatial summation [33]. As women in general have smaller muscles, this relative difference may contribute to the lower pressure pain thresholds. Another aspect is the greater degree of muscle temporal summation in women [34] which could cause increased pain integration during the duration of the pressure stimulus causing lower PPTs. Therefore, it is most likely that peripheral and central mechanisms are responsible for gender differences in PPT. Future studies investigating further the role of peripheral and central mechanisms are warranted.

### Topographical mapping of pain sensitivity

The pressure pain maps of the neck-shoulder and low back regions delineated spatial changes. On the other hand, no differences between genders were found in the normalized PPT maps. The normalization procedure of the PPT data removed the absolute differences in PPT among genders. This was done to enable the study of the spatial information contained in the PPT maps. The lower part of trapezius was less sensitive than the upper part which is in line with our previous findings [24]. This further supports the relation between the anatomical subdivisions of the muscle [35], neuromuscular activity [36,37] and sensory partitioning [25]. This gradient in pressure pain levels could also be related to the different amounts of measurement points located on muscle belly and musculo-tendinous tissue within each subdivision as musculo-tendinous parts are less sensitive to pressure pain than the muscle belly parts [22,23]. These results further support the importance of the upper part of the trapezius muscle as a major contributor to musculoskeletal pain in the neck-shoulder region [38].

The assessments along the spine did not show distinct differences in sensitivity but showed a gradual increase in PPT in the caudal direction in line with a recent study by O'Neill et al. [39]. In accordance with our results previous studies also found no difference in PPT between sides in the upper back and shoulder regions [22,27,40]. In the lumbar region of the back there were symmetrical proportions in the pain topography between the left and right erector spinae muscle, while the measurements on the spine part showed that it was far less sensitive to pressure pain than the musculo-tendinous parts. The measurements on the edge of the muscles showed to be the most sensitive, which is in accordance with previous studies investigating the lumbar region in healthy subjects [40].

### Methodological Limitations

The reliability of the PPT measurements in the present study was found to be high. This is in line with the excellent inter-examiner reliability in PPTs measured in healthy subjects [41]. The effect sizes were low for PPT differences among genders and medium to large for differences in trapezius sub-divisions and spinal processes and the left/right side of the lumbar region. This meant that these factors accounted for 2-19% of the overall variance. Thus, we should recognize that the present gender differences were minor and that greater sample sizes are required to delineate gender effects. The distance between adjacent points and the time between consecutive measurements at the same point (at least 10 minutes) prevented spatial and temporal summation [24,33]. We have also recently reported that PPT values are unchanged in healthy subjects when comparing measurements performed with intervals of 30 minutes and 24 hours [24]. However, this might not be the case in patient populations.

## Conclusions

The present study reported for the first time the spatial distribution of pressure pain sensitivity in cervico-thoracic and lumbar regions among healthy subjects. Despite the low population size and the small effect size we found that women were more sensitive than men to pressure pain stimulation in both the cervico-thoracic and the lumbar regions with no gender differences in normalized pressure pain maps. This investigation provides the basis for further clinical studies on e.g. chronic shoulder pain, low back pain or osteoporosis.

## Competing interests

The authors declare that they have no competing interests.

## Authors' contributions

ATB performed the measurements and analyzed the data. All authors contributed to the conception, design, interpretation of data, and the revision of the manuscript. All authors have read and approved the final manuscript.

## Pre-publication history

The pre-publication history for this paper can be accessed here:

http://www.biomedcentral.com/1471-2474/11/234/prepub
